# Supplementary material for: Complex‐centric proteome profiling by SEC‐SWATH‐MS
Source: Mol Syst Biol. 2019 Jan 14;15(1):e8438. doi: 10.15252/msb.20188438 (PMC6346213; doi:10.15252/msb.20188438)
Supplement: Supplementary file 7 — Dataset EV6 [file MSB-15-e8438-s007.zip › feature_plots_bioplex/P25789.pdf]

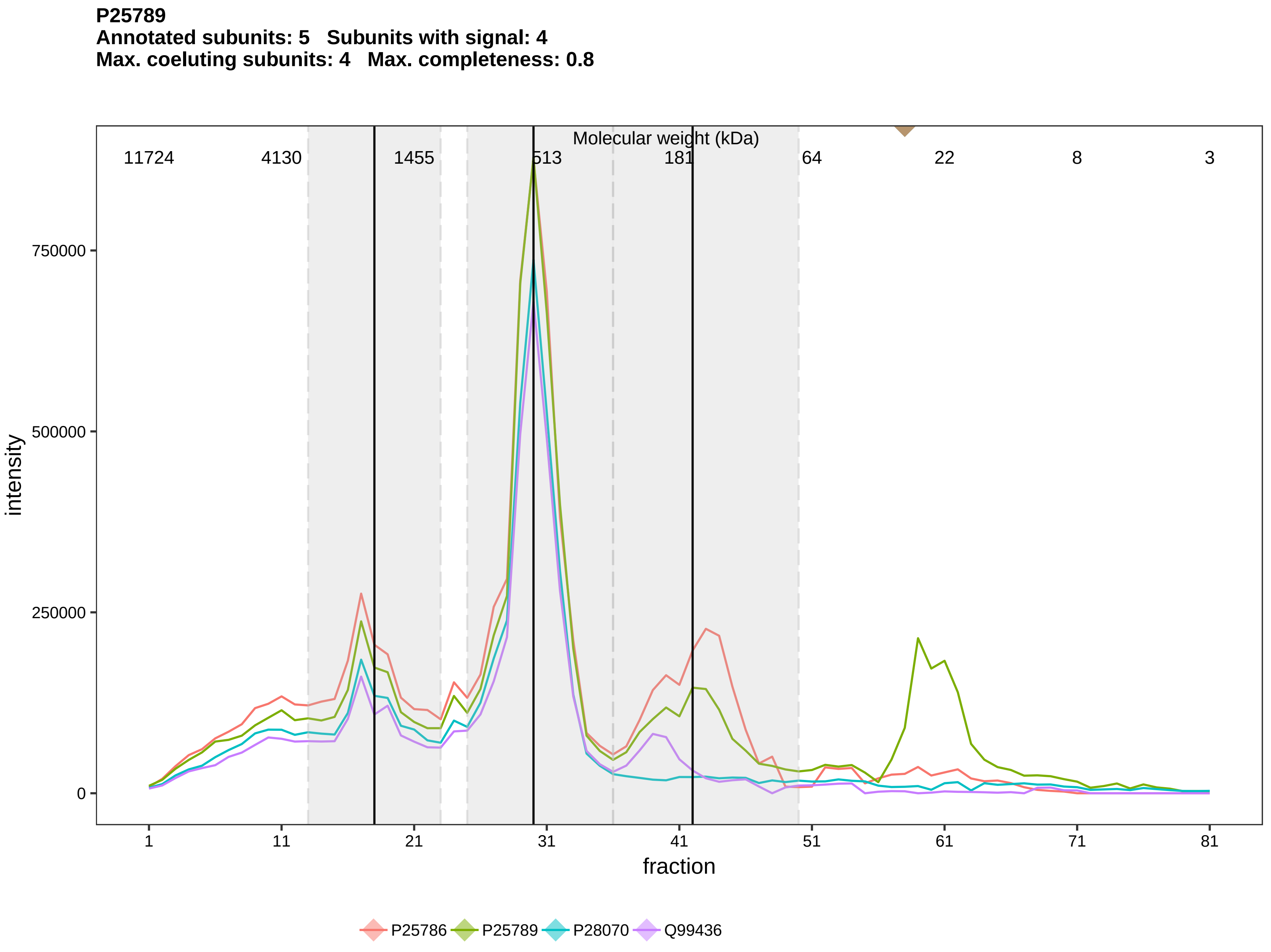
P25789
Annotated subunits: 5   Subunits with signal: 4
Max. coeluting subunits: 4   Max. completeness: 0.8
Molecular weight (kDa)
11724
4130
1455
513
181
64
22
8
3
intensity
750000
500000
250000
0
1
11
21
31
41
51
61
71
81
fraction
P25786
P25789
P28070
Q99436
